# P-1117. In-hospital cluster of Mycobacterium lentiflavum colonization/infection at a Japanese tertiary care center: outbreak investigation

**DOI:** 10.1093/ofid/ofaf695.1312

**Published:** 2026-01-11

**Authors:** Hitoshi Honda, Shushi Okuno, Junko Makino, Marie Ikai, Kotaro Sawai, Yusuke Minato

**Affiliations:** Fujita Health University School of Medicine , Toyoake, Aichi, Japan; Tachikawa Sogo Hospital, Tachikawa, Tokyo, Japan; Tachikawa Sogo Hospital, Tachikawa, Tokyo, Japan; Fujita Health University, Toyoake, Aichi, Japan; Fujita Health University, Toyoake, Aichi, Japan; Fujita Health University, Toyoake, Aichi, Japan

## Abstract

**Background:**

*Mycobacterium lentiflavum* is a ubiquitous yet rare nontuberculous mycobacterium (NTM) found in drinking water and public water systems, posing a potential contamination risk. NTMs have been increasingly reported in healthcare settings, causing pulmonary diseases. In April 2024, we identified a cluster of hospitalized patients with sputum cultures positive for *M. lentiflavum*, suggesting nosocomial transmission. An outbreak investigation was undertaken to determine the source of infection or colonization, and infection control measures were implemented.

**Figure:**
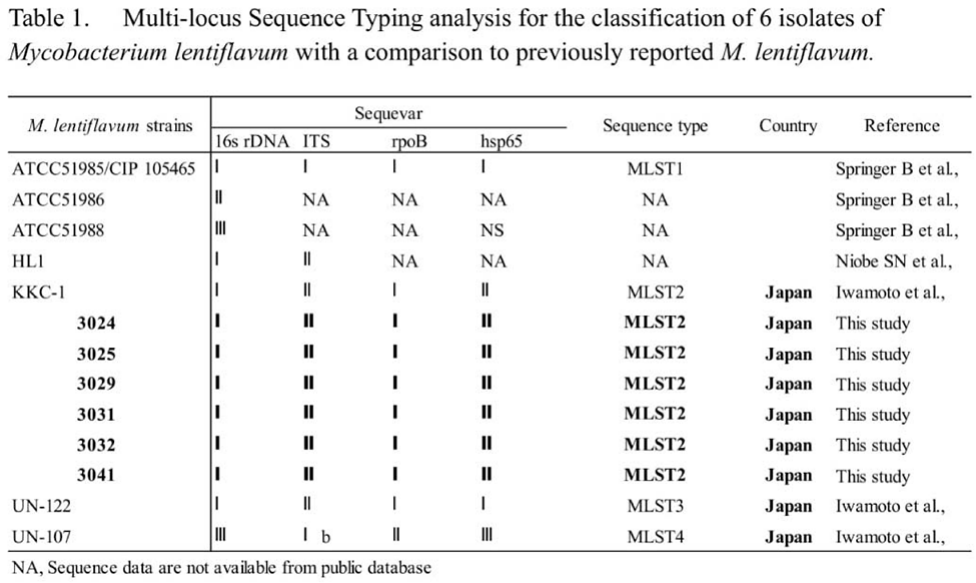
Multi-locus Sequence Typing analysis for the classification of 6 isolates of Mycobacterium lentiflavum with a comparison to previously reported M. lentiflavum

**Figure:**
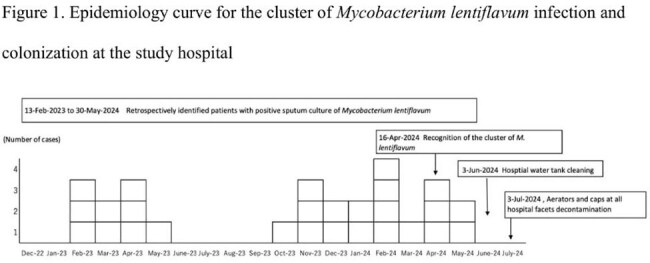
Epidemiology curve for the cluster of Mycobacterium lentiflavum infection and colonization at the study hospital

**Methods:**

The cluster occurred at Tachikawa Sogo Hospital, a 287-bed acute care hospital in Tokyo, Japan. Following the identification of an *M. lentiflavum* cluster in April 2024, patients with NTM-positive cultures from February 2023 to May 2024 were retrospectively assessed to determine if a long-standing outbreak had occurred. Environmental sampling included 113 water samples (200 mL each) collected from four-bed rooms (n=34), private rooms (n=35), nursing stations, nurse break rooms at the six hospital wards, the nutritional department, and water reservoirs. Air-conditioner outlets were also examined. Multi-locus sequence typing (MLST) was performed on selected clinical isolates to determine clonality.

**Results:**

In April 2024, a hospital infection control nurse reported *M. lentiflavum* in four hospitalized patients. A retrospective investigation (February 2023–May 2024) subsequently identified 27 cases (Figure 1), with two meeting the NTM disease criteria outlined by the ATS/IDSA guidelines. MLST of six selected isolates showed no allelic variants and 100% identity. Compared with deposited data, 16S rDNA and *hsp65* genes exhibited 100% identity with *M. lentiflavum* ATCC51985, while 16S-23S rRNA and *rpoB* genes matched *M. lentiflavum* KKC-1, previously isolated in Japan (Table 1). In May 2024, hospital faucets were thoroughly cleaned, and aerators were sterilized with sodium hypochlorite. Additional chlorination was performed in June 2024. As of September 2024, no new cases have been detected.

**Conclusion:**

The likely source of the *M. lentiflavum* cluster was contaminated hospital tap water aerators. Given that some patients were clinically infected, aggressive measures are needed to control nosocomial outbreaks.

**Disclosures:**

Yusuke Minato, Ph.D., Shionogi & Co., Ltd.: Grant/Research Support

